# Filaggrin genotype does not determine the skin's threshold to UV-induced erythema

**DOI:** 10.1016/j.jaci.2015.11.022

**Published:** 2016-04

**Authors:** Deborah Forbes, Leona Johnston, June Gardner, Stephanie F. MacCallum, Linda E. Campbell, Albena T. Dinkova-Kostova, W.H. Irwin McLean, Sally H. Ibbotson, Robert S. Dawe, Sara J. Brown

**Affiliations:** a Ninewells Hospital and Medical School, Dundee, United Kingdom; b Photobiology Unit, Dermatology Department, University of Dundee, Ninewells Hospital and Medical School, Dundee, United Kingdom; d Division of Cancer Research, Medical Research Institute, Jacqui Wood Cancer Centre, University of Dundee, Ninewells Hospital and Medical School, Dundee, United Kingdom; c Dermatology and Genetic Medicine, Division of Molecular Medicine, College of Life Sciences and College of Medicine, Dentistry and Nursing, University of Dundee, Dundee, United Kingdom; e Dermatology and Genetic Medicine, Medical Research Institute, College of Medicine, Dentistry and Nursing, Ninewells Hospital and Medical School, Dundee, United Kingdom

To the Editor:

Profilaggrin and filaggrin play multiple roles in the formation and function of the epidermal barrier, contributing to protection against dehydration, mechanical stress, infection, and, it has been proposed, photodamage.^1^ Loss-of-function mutations in the gene encoding filaggrin *(FLG)* represent the strongest and most significant genetic risk factor for atopic dermatitis (AD) identified to date.^1^ Proteolysis of filaggrin releases histidine and other amino acids into the stratum corneum. Histidine is converted by the enzyme histidase (histidine ammonia-lyase) to *trans*-urocanic acid (*trans*-UCA), which can then undergo photoisomerization on absorption of UVB to produce *cis*-UCA (see Fig E1 in this article's Online Repository at www.jacionline.org). There is experimental evidence to suggest that *cis*-UCA has immunomodulatory and photoprotective effects. The local and systemic immunosuppressive effects of *cis-*UCA were initially demonstrated in murine models, and more recently, histidinemic mice deficient in cutaneous UCA because of a mutation in *Hal*, the gene encoding histidase, have been reported to show increased propensity to UVB-induced DNA damage.^2^ Mice deficient in caspase-14 (an enzyme in the profilaggrin-filaggrin proteolytic pathway) show accumulation of cyclobutane pyrimidine dimers in response to UVB radiation and increased apoptosis in the epidermis, indicating a role for caspase-14 in UVB scavenging within the stratum corneum.^3^ The immunosuppressive effects of *cis*-UCA have been demonstrated in human keratinocytes and leukocytes *in vitro*; knockdown of *FLG* in organotypic culture results in increased susceptibility of keratinocytes to UV-induced apoptosis.^4^ Loss-of-function mutations and copy number variation in *FLG* are known to result in lower levels of filaggrin breakdown products, including UCA, in human stratum corneum. Therefore it has been postulated that *FLG* genotype might in part determine the photoprotective capacity of human skin (see Fig E1),^1^ but experimental evidence *in vivo* is lacking.

We aimed to test the hypothesis that filaggrin deficiency resulting from loss-of-function mutations in *FLG* is associated with increased erythemal sensitivity to UV radiation. Cutaneous response to UV radiation was assessed by using the minimal erythema dose (MED; the lowest dose of UV causing just perceptible skin redness) as a quantifiable surrogate end point for cutaneous damage. We used detailed monochromator phototesting of 71 adult volunteers of white European ethnicity with clinically normal skin; the demographic characteristics are summarized in Table I. A calculation performed before this study commenced indicated that 7 or 8 *FLG* mutation carriers within a total study size of 70 to 80 subjects would provide sufficient statistical power to detect a 1.8-fold difference in MED. This sample size estimation was based on known variability in MEDs from previous studies and assuming comparisons of arithmetic means of log-transformed data (therefore able to back–transform differences into fold differences). Details of the power calculation are shown in the Methods section in this article's Online Repository at www.jacionline.org.

This work was approved by the East of Scotland Research Ethics Committee (reference 14/ES/0030), and the study was conducted in accordance with the Declaration of Helsinki.

Participants were screened for the 6 most prevalent loss-of-function mutations in *FLG* in the white European population (R501X, 2282del4, R2447X, S3247X, 3673delC, and 3702delG) by using published methodology.^5^ Ten (14%) of 71 were found to be heterozygous for a loss-of-function mutation in *FLG* (Table I). Fitzpatrick sun-reactive skin phototype was recorded for 45 of 71 subjects, and no difference was detected (*P* = .14, χ^2^ test) in skin phototypes between the genotype subgroups.

Up to 7 separate wavebands from 295 to 430 nm, representing a spectrum from UVB to UVA and visible light, were tested on the 71 subjects. A detailed description of phototesting methods is given in the Methods section in this article's Online Repository. Subjects were grouped according to *FLG* genotype, and MEDs were compared by using nonparametric rank-based methods (because some MED values were greater than or less than test dose ranges) with the Mann-Whitney *U* test (see the Methods section in this article's Online Repository) to derive CIs for differences in median MEDs (see Table E1 in this article's Online Repository at www.jacionline.org). We detected no significant differences in MEDs (defined as *P* ≤ .05) between the *FLG* wild-type and *FLG* heterozygous groups at any of the wavebands tested (Fig 1 and see Table E1). The CIs for differences were sufficiently narrow to make any large differences in MEDs between the genotype groups unlikely.

It has previously been reported that AD might be associated with photosensitivity,^6^ a lower threshold to UVB-induced erythema,^7^ or both. Some epidemiologic data also suggest a higher incidence of multiple nonmelanoma skin cancers in subjects with a history of AD.^8^ Loss-of-function mutations in *FLG* are strongly associated with AD, and there is widespread downregulation of filaggrin expression in the skin of patients with atopic eczema, which has been demonstrated at the transcriptome level by means of direct RNA sequencing,^9^ and in the breakdown products of filaggrin in the stratum corneum, which was quantified by means of HPLC.^10^ A partial reduction in expression of filaggrin might result from the effect of circulating inflammatory cytokines, whereas a more profound deficiency results from loss-of-function mutations in *FLG* leading to near-complete absence of profilaggrin in the homozygous or compound heterozygous state. Therefore it can be hypothesized that filaggrin deficiency contributes to the observed photosensitivity and/or reduced threshold to UVB-induced erythema in patients with AD. We have performed a detailed analysis of cutaneous photoresponse in clinically normal skin to avoid the confounding effects of atopic inflammation. Our findings have excluded a large effect of *FLG* genotype on photosensitivity (≥1.8-fold difference in MED) at any of the wavebands tested. In addition, the results of our monochromator phototesting did not indicate a differential erythemal sensitivity within the wavelengths representing UVB, as would be predicted from the known absorption spectrum of UCA.

One limitation of our study is that the healthy volunteers did not include any subjects with ichthyosis vulgaris, and therefore we have not excluded the possibility that *FLG* homozygous (or compound heterozygous) subjects might show greater erythemal sensitivity than wild-type subjects. However, *FLG*-null heterozygosity has a significant effect on filaggrin expression *in vivo*,^9^, ^10^ and therefore we would expect an effect to be observed in *FLG* heterozygotes if this was substantial.

The fact that observations of UVB-induced damage in murine and *in vitro* models have not been supported by clinical data suggest that different mechanisms lead to cutaneous erythema *in vivo* than the markers of UV damage studied *in vitro* and in mice. For example, apoptosis is known to occur within areas of skin damaged by UV exposure, and this is associated with cutaneous erythema, but the relationship is nonlinear. Furthermore, the photoprotective effect of the *FLG* wild-type genotype might be attributable to a mechanical filtering of UV radiation by the stratum corneum rather than by chemical photoimmunosuppression.

In conclusion, our *FLG* genotype–stratified analysis of responses to UV and visible radiation in clinically normal skin does not support the hypothesis that the breakdown products of filaggrin play a major role in the sensitivity of human skin to UV-induced erythema. This has relevance to the ongoing search for predictors of patient response in phototherapy for AD and for the development of personalized medicine.

## Figures and Tables

**Fig E1:**
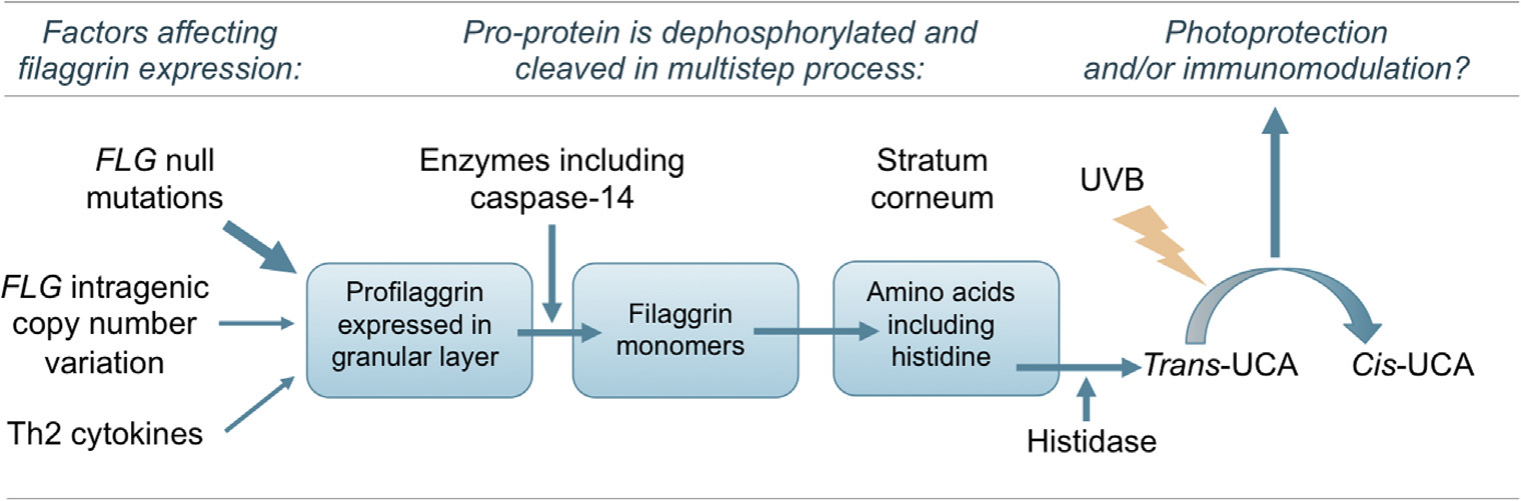
Diagrammatic summary of factors affecting the profilaggrin–*cis*-UCA pathway Previous studies have demonstrated the effects of variation within *FLG*E6, E7, E8 and levels of T_H_2 cytokinesE10, E9 on filaggrin expression. The role of caspase-14 in profilaggrin processing has been illustrated in mice.^E11^ Filaggrin is degraded to release a pool of amino acids rich in histidine^E12^ in the stratum corneum, contributing to barrier function through hydration and acidification.^E13^ The conversion of histidine to *trans-*UCA is catalyzed by histidase,^E14^ and the *trans-*isomer is converted to *cis-*UCA by UVB.^E15^ UVB absorption might contribute to cutaneous photoprotection, and *cis-*UCA might have additional immunomodulatory effects.E16, E17, E18, E19, E20

**Table E1:** Results of monochromator phototesting of 71 healthy volunteers stratified according to *FLG* genotype

Waveband	295 ± 5 nm	300 ± 5 nm	305 ± 5 nm	335 ± 30 nm	365 ± 30 nm	400 ± 30 nm	430 ± 30 nm
Median MED (mJ/cm^2^)						
*FLG* wild-type subjects	10.0 (n = 53)	18.0 (n = 53)	47.0 (n = 61)	5,600 (n = 61)	24,000 (n = 61)	>82,000 (n = 61)	>82,000 (n = 61)
*FLG* heterozygous subjects	8.2 (n = 8)	15.0 (n = 8)	47.0 (n = 10)	4,950 (n = 10)	20,000 (n = 10)	82,000 (n = 10)	>82,000 (n = 10)
Difference	1.8 mJ/cm^2^ higher in *FLG* wild-type	3.0 mJ/cm^2^ higher in *FLG* wild-type	No difference	650 mJ/cm^2^ higher in *FLG* wild-type	4,000 mJ/cm^2^ higher in *FLG* wild-type	Not quantifiable	Not quantifiable
95% CI for difference	−2.6 to 5.0	−3.0 to 7.0	−9.0 to 14.0	−1,200 to 2,300	−7,000 to 9,000	Not quantifiable	Not quantifiable
*P* value (Mann-Whitney *U* test)	.41	.35	.79	.62	.74	.58	.70

Some cells are “not quantifiable” because results of greater than the highest test dose were recorded.

**Fig 1:**
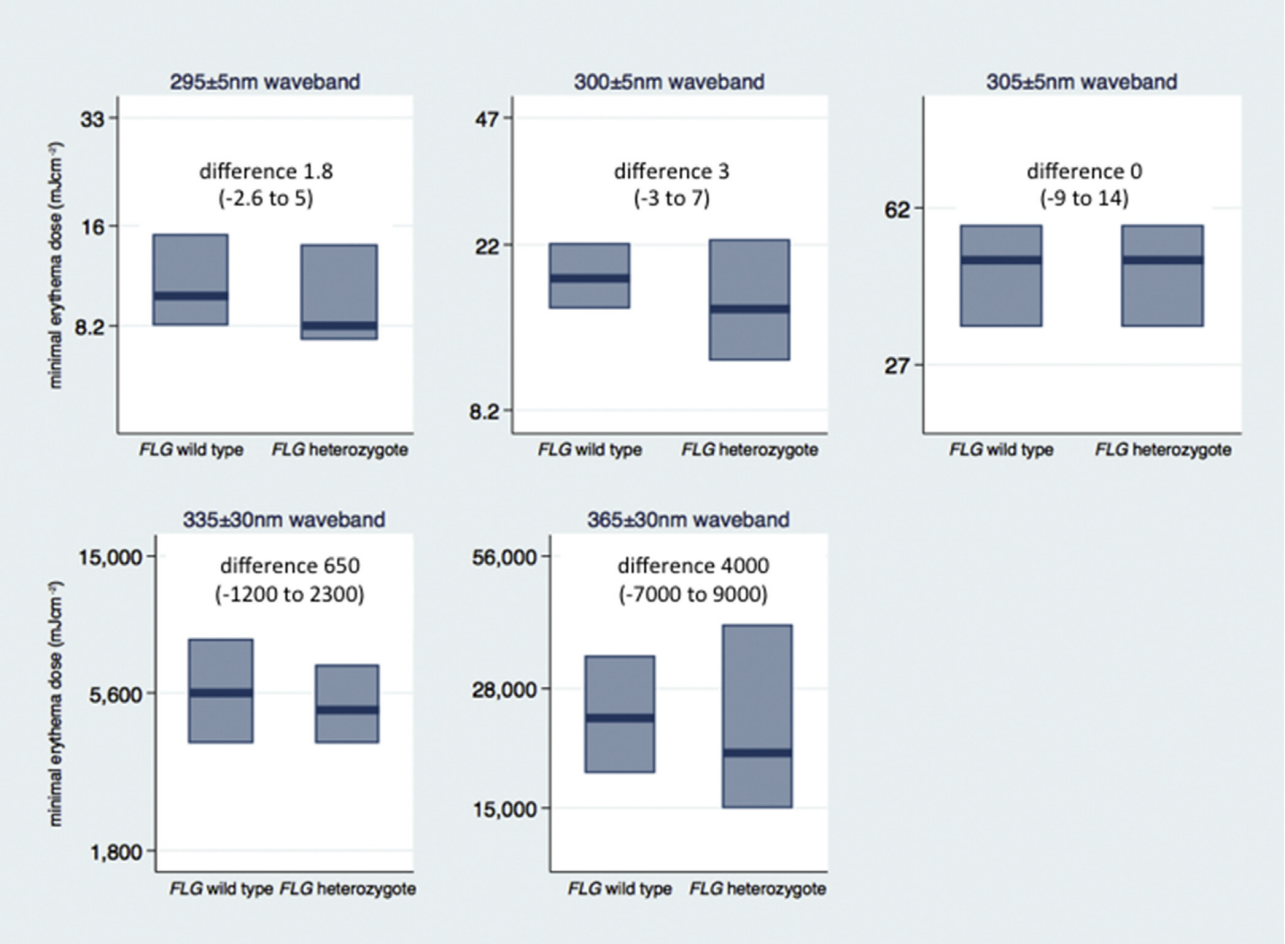
Box plots showing monochromator phototesting MED results in healthy volunteers of different *FLG* genotypes. The findings for 5 distinct wavebands are shown. Results for the 400 ± 30 and 430 ± 30 nm wavebands showed no difference between *FLG* wild-type and heterozygotes (see Table E1); these data are not displayed because the median MEDs and ranges are not quantifiable. *Boxes* indicate interquartile ranges, and the *bar within each box* marks the median result. The difference in median MEDs (and 95% CIs) are shown above each plot. All values are in millijoules per square centimeter. Median MEDs were compared by using the Mann-Whitney *U* test. There were 61 *FLG* wild-type subjects and 10 *FLG* heterozygous subjects tested in each group, with the exception of the 295 nm and 300 nm wavebands, in which data were obtained on 53 *FLG* wild-type subjects and 8 *FLG* heterozygous subjects.

**Table I:** Demographic data and *FLG* genotype results for 71 volunteers with clinically normal skin

Sex	43 male/28 female
Age (y), range (median)	22-70 (41)
*FLG* wild-type subjects (no.)	61
*FLG* heterozygotes (no.)	10
Total (no.)	71

Volunteers were screened for the 6 most prevalent *FLG* loss-of-function mutations in the population. Five subjects were heterozygous for R501X, 3 were heterozygous for 2282del4, 1 was heterozygous for R2447X, and 1 was heterozygous for S3247X. No 3673delC or 3702delG mutations were detected, and there were no homozygotes or compound heterozygotes. Fitzpatrick skin phototype was recorded for 45 of 71 subjects, and there was no significant difference (*P* = .14, χ^2^ test) in skin phototypes between the genotype subgroups.

## Methods

### Assessment of MEDs of different wavebands in healthy volunteers

Healthy adults with clinically normal skin of phototypes I to III^E1^ who had previously participated in research studies within the National Photobiology Unit (Dundee, Scotland, United Kingdom) were invited to participate in this study. Exclusion criteria were history of skin allergy, eczema/AD, psoriasis or polymorphic light eruption, immunosuppression caused by medications or disease, photosensitizing medication, and a holiday abroad or sunbed use within the preceding 4 weeks. A total of 71 subjects provided written informed consent and a saliva sample for DNA extraction. Emollient application to skin test sites was not allowed within the 48 hours before phototesting.

Detailed phototesting was undertaken by using an irradiation monochromator, which is a diffraction grating device with a 1.6-kW or 450-W xenon arc lamp.E2, E3 This instrument allows irradiation of small areas of the skin over a range of wavebands, which are included in the solar spectrum (UVB to UVA/visible). Monochromator phototesting was performed as follows. On day 1, approximately 1-cm^2^ areas of the skin on the volunteer's back were exposed to UV and visible light according to a standardized procedure established in the National Photobiology Unit. A range of doses of UV and visible light at specific narrow wavebands was used for all subjects centered on (with half-maximum bandwidth) 295 ± 5, 300 ± 5, 305 ± 5, 335 ± 30, 365 ± 30, 400 ± 30, and 430 ± 30 nm.

The MED, which was defined as the minimum dose producing just perceptible erythema for each waveband tested, was determined 24 hours after irradiation. The irradiation procedure was repeated on day 2 on a separate area of back skin using smaller dose increments (10% to 20%) across a narrower range of doses at each waveband selected on the basis of the MEDs seen 24 hours after first irradiation to establish the MED precisely. Final MEDs were assessed 24 hours later (day 3).

### Power calculation

Our prestudy sample size calculation was performed to determine how many subjects were likely to be needed to detect a clinically important difference in MEDs between the *FLG* genotype groups within the 305 ± 30 nm waveband. MED data derived from testing with a geometric dose series do not follow a normal distribution, and therefore we based our sample size on the minimum difference in arithmetic means of natural log-transformed MEDs. This method was used because differences in arithmetic means of log-transformed data can be “back-transformed” to fold differences (eg, 1.8-fold), which is more understandable than the difference in log-transformed MEDs that equates to this.

MEDs for the 305 ± 5 nm waveband (representing a narrow waveband in the UVB region, the waveband of interest) from 120 healthy volunteers tested at the National Photobiology Unit were used to derive variance. These nonnormal data were log-transformed, and the numbers needed to detect a 1.5-, 1.8-, and 2-fold difference in geometric means were estimated by using Stata 12.1 software (StataCorp, College Station, Tex). Assuming that approximately 10% of the study participants would have 1 or more *FLG* loss-of-function mutations,^E4^ to obtain an 80% power with a *P* value of .05, we required at least 7 or 8 subjects with *FLG* mutations (expected within a total sample size of 70-80 subjects) to detect a difference of 1.8-fold in mean natural log-transformed MEDs at this waveband between the wild-type and *FLG*-null groups. Similarly, 55 volunteers, including 5 or 6 *FLG* mutation carriers, were needed to detect a 2-fold difference in mean natural log-transformed UVB MEDs.

Therefore the results arising from our sample size, including 71 volunteers and 10 *FLG* mutation carriers, have sufficient statistical power to effectively exclude an association with *FLG* genotype and erythemal response of a 1.8-fold or greater difference at the wavebands tested.

### Statistical analysis of monochromator phototesting data

When analyzing our data, we used nonparametric methods reliant on ranks, rather than absolute values, instead of parametric methods based on transformed data. This was for the practical reason that although we obtained ranks for all MEDs, the precise values for some were unknown (greater than or less than our test dose range). Some MEDs were determined to be greater than or less than the test irradiation ranges. A small number was added to all MEDs at greater than the top dose tested for wavebands of 305 nm and longer and a small number was subtracted from MEDs of less than the lowest dose tested to allow appropriate rank-based analyses. The phototesting results were compared with *FLG* genotype status (*FLG* wild-type or *FLG* mutant) by using the Mann-Whitney *U* test to analyze these nonparametric data to test the null hypothesis that there was no association of MEDs with genotype. Corresponding CIs around medians were derived by using the methods of Altman and Gardner.^E5^ We took a *P* value of .05 or less to be significant.
